# Surgical Excision of Palisaded Neutrophilic and Granulomatous Dermatitis of the Vulva: A Case Report

**DOI:** 10.7759/cureus.64965

**Published:** 2024-07-20

**Authors:** Mahmoud A Elhendawy, Ahmed M Omran, Abdulkarim Hasan, Mostafa Basiony, Ayman Abdelmaksoud, Samah S Elbasateeny

**Affiliations:** 1 Plastic Surgery, Faculty of Medicine, Al-Azhar University, Assiut, EGY; 2 Plastic Surgery, Faculty of Medicine, Al-Azhar University, Damietta, EGY; 3 Pathology, Faculty of Medicine, Al-Azhar University, Cairo, EGY; 4 Dermatology, Venereology and Leprology Hospital, Mansoura, EGY; 5 Pathology, King Abdulaziz University Faculty of Medicine, Rabigh Branch, Jeddah, SAU; 6 Pathology, Zagazig University, Zagazig, EGY

**Keywords:** infection immunology, complete excision, vulvar reconstruction, systemic lupus erythematosus, palisaded neutrophilic granulomatous dermatitis

## Abstract

Palisaded neutrophilic and granulomatous dermatitis (PNGD) is an inflammatory cutaneous disorder of unknown etiology that typically occurs in association with systemic disease. Rheumatoid arthritis and systemic lupus erythematosus are the most common associated diseases. PNGD manifests as skin-colored to erythematous papules and plaques, mainly on the extremities. However, to the best of our knowledge, no cases of PNGD in the vulva have been reported in foreign literature to date. Herein, we report the first case of a 31-year-old female with systemic lupus erythematosus disease who presented multiple plaques and a pigmented, rough, mamillated skin surface affecting the vulva, leading to disfigurement of the vulva and interfering with sexual intercourse due to severe pain, irritation, and frequent infection. Surgical excision of the whole lesion with reconstruction of the vulva was done in two sessions and histologically diagnosed as PNGD.

## Introduction

Palisaded neutrophilic granulomatous dermatitis (PNGD) is a rare, benign skin disorder, recently considered a type of granulomatous dermatitis (GD). This reactive inflammatory dermatosis is highly associated with systemic disorders and is characterized by distinct histopathological characteristics and clinical manifestations [1,2].

The exact cause of this reactive dermatosis is poorly understood. However, its occurrence in association with systemic inflammatory disorders raises the possibility that the dysfunction of the immune system may play a central role in its pathogenesis. The precipitation of immune complexes in small blood vessels of the skin in association with a systemic disorder or due to an external cause, such as drugs, can trigger an inflammatory granulomatous reaction with collagen damage [1]. This reaction results in the formation of papules, with many secondary morphological changes such as erosion, yellowish crusting, umbilications, or central perforation [3]. The PNGD may also present as erythematous or violaceous papules, annular plaques, or nodular lesions [4].

PNGD may occur in any age group but is rare in children. It affects women about three times more than men, reflecting the distribution of associated systemic disorders [5,6].

PNGD is a relatively uncommon lesion that could be misdiagnosed or underdiagnosed [3], and despite the benign nature of the lesion, the diagnosis is essential because PNGD is often a dermal manifestation of an underlying systemic disorder. Skin biopsy is the gold standard for the diagnosis of PGND, and treatment is mainly targeted at the underlying disorder [6].

## Case presentation

**Figure 1:**
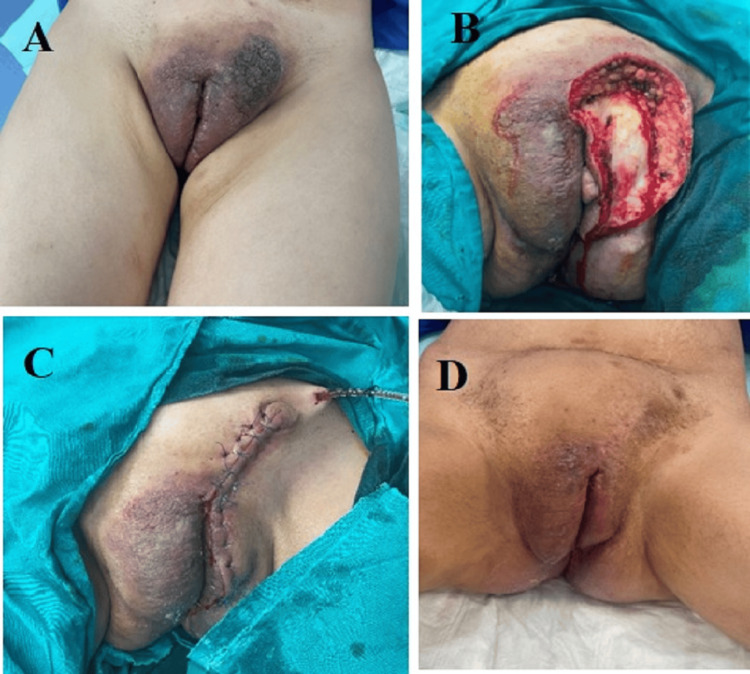
The first surgery A) The lesion before surgery. B) Excision. C) Immediate postoperative following reconstruction by a local advancement flap. D) Late postoperative after complete good healing.

A 31-year-old female presented with a three-year duration of tender, erythematous papules involving the vulva and extending down to the perineum, which continued to wax and wane. The lesion became pigmented and had a rough, mamillated surface with frequent infection on top of the lesion associated with translucent discharge. The patient suffered from severe disfigurement at the vulva (Figure 1), with frequent infections associated with an inability to perform sexual intercourse. The patient had a significant medical history of systemic connective tissue disease suspected to be systemic lupus 10 years ago, associated with ulcerative colitis. Our plan for the management of this severe disfigurement of the vulva was surgical excision of the lesion and reconstruction in two stages: the first was an excisional biopsy to the left half of the vulva, and after confirmation of the diagnosis, a whole excision of the rest of the lesion of the vulva with reconstruction was performed.

Surgical procedure: The patient prepared for the first stage by doing all routine investigations in the form of a CBC, kidney function test, liver function, prothrombin time (PT), platelet count (PC), and international normalized ratio (INR). All investigations were normal. Three days before the operation, the patient washed the region of the lesion daily with a betadine scrub to minimize infection. At the time of operation, marking was done to determine the excised part of the vulva and to design the method for reconstruction.

**Figure 2:**
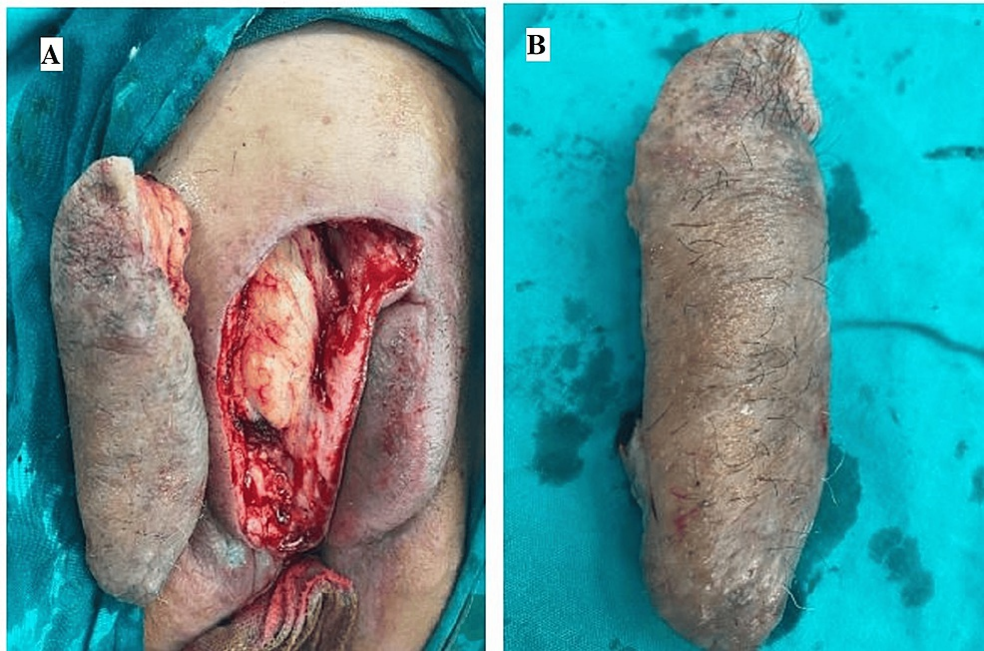
The second surgery A) The soft tissue defect and the excised part involving the lesion. B) The excised part

The patient underwent spinal anesthesia and lay down in a supine position. The left half of the vulva was excised and reconstructed by an advancement flap from the suprapubic region (Figure 2).

**Figure 3:**
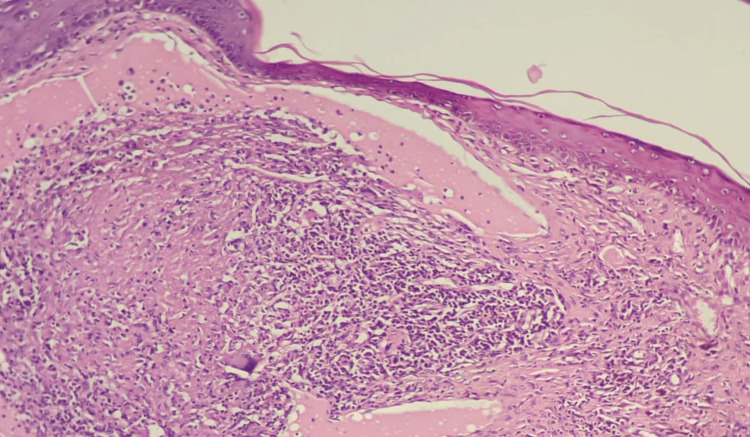
Histological features of the excised lesion Epidermal covering with the underlying lesion showing degenerating collage and a diffuse, dense granulomatous inflammatory infiltrate including histiocytes, neutrophils, and occasional multinucleated giant cells throughout the dermis (H&E stain, original magnification 40×)

The specimen was taken and a histopathological examination was performed, which showed keratosis with focal parakeratosis, a focally thickened granular cell layer, mild spongiosis, epidermal hyperplasia, and inflammatory infiltration within the epidermal basal and squamous cell layers (Figure 3).

The dermis showed a marked superficial and deep perivascular mixed inflammatory infiltrate composed predominantly of neutrophils, lymphocytes, plasma cells, histocytes, and giant cells with a focal interface pattern. The dermal papillae showed edema and fibrosis with collagen deposition in a vertical orientation perpendicular to the epidermis. Features of vasculitis-like changes were also seen, in addition to dermal mucin surrounded by chronic inflammatory cellular infiltrate.

The provisional diagnosis was a pigmented vulvar skin lesion for excision. On an excisional biopsy, the microscopic picture is consistent with palisaded neutrophilic granulomatous dermatitis on top of active chronic inflammation of a suggested autoimmune nature with secondary dermal mucinosis and lichen simplex chronicus.

After three months and confirmation of diagnosis by histopathology, the patient was prepared for second-stage surgery. The rest of the lesion on the right side of the vulva was completely excised and reconstructed by an advancement flap, resulting in a complete reconstruction and restoration of the aesthetic appearance of the vulva.

Postoperative medical treatment in the form of antibiotics for one week (ceftriaxone 1 mg once daily) and analgesia (acetaminophen 500 mg every 6 hours). A daily wash of the genitalia with betadine scrub for two weeks postoperatively was recommended. Follow-up was done for six months with complete healing of all wounds and a very nice aesthetic appearance of the vulva without recurrence of the lesion.

## Discussion

PNGD is a rare dermatological response to underlying systemic and autoimmune disorders, mostly rheumatoid arthritis, connective tissue disease, and lymphoproliferative disorders [5]. It is also reported to occur in association with systemic lupus erythematosus, inflammatory diseases of the bowel, myelomas, cancers, infection, and some drugs [7]. Females are more likely to be affected, reflecting the incidence of systemic diseases linked to PNGD development [8,9].

Thus, PNGD is clinically regarded as an early indicator of a variety of systemic disorders. Similar to previously reported cases of PNGD, our patient is a female with a history of many years of suffering from SLE and UC.

Clinically, the PNGD presented as painful or asymptomatic, with multiple umbilicated papules showing crust formation or central perforation. It is sometimes detected as erythematous or violaceous papules, nodular lesions, and annular plaques. It less commonly presents as petechiae [4]. PNGD is usually detected on the extensor surfaces of limbs, especially the elbows and fingers, or symmetrically on the face and trunk. Occasionally, the lesion appears on the flank as linear elongated cords, named a “sign of burning rope” [5]. Tajima et al. reported a rare presentation of PNGD in the form of pustules in a woman with SLE [10].

In our report, we described a rare case of PNGD in a woman with SLE, involving the vulva and extending down to the perineum. To the best of our knowledge, this is the first instance of such a PNGD presentation.

Biopsies of PNGD are variable histologically depending on the age and stage of the lesions. Early PNGD lesions demonstrate leukocytoclastic vasculitis of small dermal blood vessels and infiltration of the dermis with lymphocytes, histiocytes, and plentiful neutrophils, mainly around areas of degenerated dermal collagen. Well-established lesions demonstrate palisaded granulomas and neutrophilic infiltration of all dermal layers that may extend to the subcutis. Late lesions contain granulomas, degenerated collagen fiber, neutrophil debris, and fibrosis. Occasionally, the granulomas are encircled by collagen and mucin, like granulomas annulare [11,12]. Recently, it was concluded that multiple biopsies from a single PNGD patient may demonstrate different histologic features [12], and this was similar to our described PNGD case.

## Conclusions

PNGD lesions display a wide spectrum, both clinically and histologically. In addition to their relative rarity, this emphasizes that the diagnosis of PNGD should always be based on clinicopathological correlation, and surgical removal of extensive lesions can effectively improve symptoms and address cosmetic concerns. Follow-up of our case and all reported PNGD cases is mandatory to control and/or manage the underlying systemic disorders, stop a specific type of medication, or detect an emergent malignancy.
